# Dietary diversity and associated factors among households and children in internally displaced person camps in Southern Somalia: A cross‐sectional study

**DOI:** 10.1111/mcn.13707

**Published:** 2024-07-31

**Authors:** Mohamed K. Ali, Lars Berglund, Renée Flacking, Munshi Sulaiman, Fatumo Osman

**Affiliations:** ^1^ School of Health and Welfare Dalarna University Falun Sweden; ^2^ Research Evaluation, Accountability, Learning and Monitoring (REALM) Save the Children International Somalia Country Office Mogadishu Somalia; ^3^ Department of Public Health and Caring Sciences, Geriatrics Uppsala University Uppala Sweden; ^4^ Epistat AB Uppsala Sweden; ^5^ Present address: Rural Development Unit, Food and Agriculture Organisation of the United Nations (FAO) Somalia Country Office Aden Adde International Airport Garowe Somalia; ^6^ Present address: BRAC Institute of Governance and Development (BIGD) BRAC University Mohakhali Dhaka Bangladesh

**Keywords:** antenatal care, child dietary diversity score, household decision making, household dietary diversity score, IDPs, Somalia

## Abstract

The study aimed to assess household and child dietary diversity in Southern Somalia by identifying determinants of adequate dietary diversity in three internally displaced person (IDP) camps in Baidoa, Dayniile and Dharkanley. A total of 1655 female main caregivers with 2370 children (6–59 months old) were included. Data on household dietary diversity score and child dietary diversity score indicators were collected from all households. The questionnaire was read face‐to‐face to the female main caregivers. Multivariate logistic regression analysis was performed to identify factors associated with adequate dietary diversity, which was defined as the consumption of at least four food groups within 24 h before the survey. The proportion of households achieving adequate HDDS was high in all locations 95.8%, 96.9% and 89.0% in Baidoa, Dharkanley and Dayniile, respectively, and the total adequate household dietary diversity score (AHDDS) was 95.6%. The proportion of adequate child dietary diversity score (ACDDS) was achieved in 63.5%, 8.5% and 38.3%. The main factors associated with AHDDS were larger household size, greater wealth, attendance of antenatal care (ANC) and joint decision‐making between husband and wife, while factors associated with ACDDS included ANC attendance, age, the consumption of ready‐to‐use therapeutic food and deworming tablets. These findings can guide future programmes and policies aimed at improving maternal and child nutrition in IDP camps in Somalia. By tackling these diverse factors, a promising pathway emerges to enhance the nutritional welfare of both households and children in IDP camps.

## Key messages

- We found a discrepancy between household and child dietary diversity scores in internally displaced person camps in Southern Somalia, while household scores were high, child scores were low. Bridging this gap is crucial for translating varied diets into improved child nutrition outcomes.
- Larger household size, wealth and joint decision‐making between male and female adults in the household impact dietary diversity. Developing targeted nutrition education can promote better nutrition.
- Given the benefits of antenatal care for both maternal and child dietary diversity, maternal nutrition education during antenatal visits should cover breastfeeding and complementary feeding choices, thus enhancing child nutrition. Timely complementary feeding, ready‐to‐use therapeutic food use and deworming treatments are also linked to improved dietary diversity. With these insights, policymakers can create comprehensive interventions for early feeding, maternal health and child nutrition.

## Graphical abstract

The study examined dietary diversity in three Somali internally displaced person (IDP) camps, involving 1655 women and 2370 children. It was found that most households had diverse diets, but children's diets were less varied. Factors like household size, maternal health care and parental decision‐making improved dietary variety, highlighting ways to enhance nutrition in IDP settings.

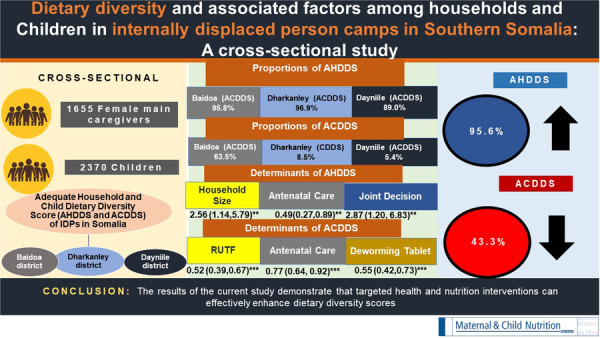

## 1 INTRODUCTION

Malnutrition is a serious concern in low‐income countries. Several factors have been shown to contribute to undernutrition, including medical conditions, infectious diseases and insufficient energy intake (UNICEF & World Health Organization, 2017). Monotonous low‐quality diets are the norm in low‐resource settings across the globe (Daniels, 2009), and the risk of various micronutrient deficiencies is high when grain‐ or tuber‐based staple foods dominate and vegetable, fruit and animal‐source food intake is limited (Abegaz et al., 2018). In settings occupied by internally displaced persons (IDPs), people face a particular risk of food insecurity, malnutrition and disease. IDPs are often supported by humanitarian and development partners who aim to reduce food and nutrition insecurity through programmes and interventions. IDPs typically reside in accommodations that lack regulation and are primarily established by landowners who create settlements to accommodate IDPs and attract humanitarian agencies. In Somalia, a substantial portion of IDPs are women and children who consistently face malnutrition, limited access to safe drinking water and the potential for outbreaks of cholera, diarrhoea and measles (Yarnell & Thomas, 2017). This is indicative of a broader governance failure to address the root causes of food insecurity and displacement (Thalheimer et al., 2023). Baidoa IDPs, often referred to as the epicentre of internal displacement due to drought and conflict, exhibit a high reliance on humanitarian aid, with households predominantly led by single women or elderly members, reflecting the severe impact of migration and conflict on traditional family structures. In contrast, Dayniile and Dharkanley IDPs face challenges related to overpopulation, inadequate infrastructure and limited economic opportunities, exacerbating the poverty levels (Ali et al., 2022; Oh et al., 2024). The household dynamics within these contexts are marked by a resilience born out of necessity, where communal and extended family support systems play critical roles in survival (Yuen et al., 2022). Similarly, the Somalia Humanitarian Response Plan indicated that more than 320,000 acutely malnourished children were in need of urgent nutrition support, including life‐saving treatment for more than 50,000 who were severely malnourished in 2017 (OCHA, 2017).

Nutrition assessments conducted in various regions of Somalia have shown that global acute malnutrition is present in 11%–15% of the population, which is above the United Nations emergency threshold of <10% (Donkor et al., 2022; Kinyoki et al., 2017; Di Marcantonio et al., 2020). Findings from the same study setting, Di Marcantonio et al. (2020), revealed that malnutrition was more prevalent in the 6–24 month age group than in the 25–59 month age group. As this high prevalence of acute malnutrition is related to multidimensional factors, policymakers are seeking integrated programme approaches to prevent the deterioration of nutrition outcomes (Singh et al., 2021). One way to understand the multifaceted nature of maternal and child undernutrition is to measure household dietary diversity and children's dietary diversity (Ali et al., 2013). Household dietary diversity, which has been measured in a range of countries as a proxy indicator of access to the food component of food security (Jones et al., 2013; Kennedy et al., 2010; Mazenda & Mushayanyama, 2021), refers to the relative composition of the number of foods or food groups consumed over a given reference period (Abdullahi et al., 2023; Bitew et al., 2021; Doocy et al., 2020; Hassan et al., 2018; Mehraban & Ickowitz, 2021; Moroda et al., 2018). Globally, various determinants have been associated with household dietary diversity, such as household decision‐making, socioeconomic status and educational levels (Amugsi et al., 2016; Di Marcantonio et al., 2020). However, there is limited understanding of the determinants associated with household and children's dietary diversity in Somalia's IDP setting. Thus, the aim of this study was to describe household and children's dietary diversity in IDP settings and their associated factors. Specifically, the research question was which factors are associated with adequate household and child dietary diversity?

## 2 MATERIALS AND METHODS

### 2.1 Study design and setting

This cross‐sectional study was conducted in 2017 in three IDP camps located in Baidoa of the Bay region and Dayniile and Dharkanley of the Banadir region, Southern Somalia. The three districts host the largest number of IDPs in Somalia, with an estimated total of 1.5 million people. Despite the high production of cereal crops and practice of raising livestock in Baidoa, its population still relies on rainfed agriculture. Without consecutive seasons of rain, its communities experience drought‐related food insecurity (Majid & McDowell, 2012). However, the population in both Dayniile and Dharkanley districts rely on the availability of food in the capital of Somalia, Mogadishu.

### 2.2 Population and sampling procedure

The Dayniile, Dharkanley and Baidoa IDP districts were purposively selected in the first stage of a two‐stage sampling procedure. These three districts have a large number of displaced communities with elevated levels of poverty and high malnutrition and were receiving support from Save the Children International (SCI) (Save the Children Somalia, 2017). The total number of households in Dayniile district was 24,000, and a sample of 136 households was selected. In Dharkanley district, there were 232,000 households, and a sample of 573 households was selected, while in Baidoa district, there were 263,000 households, and a sample of 946 was selected. The sampling interval was calculated by dividing the number of households in each IDP camp by the desired sample size in the camp. A list of all households in a camp was created, and a systematic random sample between 1 and 176 was chosen. Female main caregivers mainly mothers, grandmothers or female relatives for clarity were the main respondents, and those who were seriously ill and/or had children with severe malnutrition were excluded as they needed to be referred for immediate health care. To check whether the sample size of 1655 enabled an estimation of household dietary and child dietary diversity, we used a single population proportion formula to calculate the minimum sample size (Lwanga et al., 1991). Accordingly, with an 80% prevalence of inadequate dietary diversity, our sample implies that the margin of error of a 95% confidence interval is 2.2%, taking into consideration the design effect with two‐stage sampling.

### 2.3 Data collection process

The data was collected using the Infant and Young Child Feeding questionnaire (WHO, 2017), which includes household dietary diversity (HDDS) and child dietary diversity (CDDS) scores adopted from the Food and Agriculture Organization of the United Nations (FAO) guidelines (Kennedy et al., 2011). Twenty data collectors were recruited through SCI's offices in Mogadishu and Baidoa. The data collectors, who were all experienced in data collection methods, received 2 days of training before commencing their work for this study. The training included how to administer the questionnaire using mobile data collection (KoBo Collect application). The data collectors read the questionnaire face‐to‐face to female main caregivers at their IDP homes. The questionnaire used for data collection was pretested and included questions on socioeconomic status and nutritional status. The questionnaire was prepared in English, translated into Somali and then translated back into English by language translators to check for consistency.

### 2.4 Measurements

#### 2.4.1 Household and child dietary diversity scales

Respondents were asked to recall all the foods and beverages they had consumed in the 24 h before the interview. A scale of the following 12 food groups was used to assess the respondents' dietary diversity: (1) cereals; (2) roots and tubers; (3) vegetables; (4) fruits; (5) meat, poultry and offal; (6) eggs; (7) fish and seafood; (8) pulses, legumes and nuts; (9) milk and milk products; (10) oils and fats products; (11) sweets and fats and (12) spices, condiments and beverages. A single point was allocated to each food group consumed in the previous 24 h, giving a maximum of 12 points on the HDDS (FAO, 2007). Similarly, to estimate dietary diversity for children, the respondents were asked to recall all the foods eaten by their children aged 6–59 months in the 24 h before the interview. A scale of the following eight food groups was used: (1) grains, roots and tubers; (2) legumes; (3) dairy products; (4) meats; (5) eggs; (6) vitamins; (7) fruits and (8) oils and fats. A single point was allocated to each of the food groups consumed, giving a maximum of eight points on the CDDS.

A binary indicator variable of adequate household dietary diversity score (AHDDS) was created from the HDDS. If members of the household consumed at least four food groups within 24 h before the survey, they were defined as AHDDS = 1; if they did not, they were defined as AHDDS = 0 (Kennedy et al., 2011). Similarly, an indicator variable of adequate child dietary diversity score (ACDDS) was created from the CDDS (Dinku et al., 2020), by which children who consumed more than four food groups were considered to have ACDDS = 1 and those who did not were considered to have ACDDS = 0.

#### 2.4.2 Wealth index

A wealth index was used as a proxy measure for the socioeconomic status of households and was based on a composite measure of household assets using principal component analysis (Shaukat et al., 2020). These household assets comprised number of bedrooms; types of roofing, wall and floor materials; drinking water sources; ownership and type of toilet and ownership of assets such as a radio, TV, mobile, refrigerator, charcoal stove, wheelbarrow, kerosene lamp, bed, sofa and car.

#### 2.4.3 Demographics and health variables

This study used the independent variables of the place of residence (Baidoa, Dharkanley and Dayniile), female main caregiver age (15–24, 25–34, 35–44 and >45 years), marital status of the female main caregiver (divorced/separated and married), household size (<6 members and ≥2 members), number of under‐5 children (<2 children and ≥2 children), literacy status of the female main caregiver (can read and write and cannot read and write) and household decision‐making about daily household purchase needs (female main caregiver, husband, joint decision‐making, someone else).

Furthermore, independent variables include antenatal care (ANC) attendance of female main caregiver (yes and no), child's age (6–11, 12–13, 24–35, 36–47 and 48–59 months), sex of the child (male and female), timely initiation of complementary feeding (yes and no), child lives with female main caregiver (yes and no), child has received deworming tablet (yes and no), child's admission to community‐based management of acute malnutrition (yes and no), child has been fed with ready‐to‐use therapeutic food (RUTF) (yes and no) and child has been sick in the last 14 days (yes and no).

### 2.5 Statistical analysis

AHDDS and ACDDS (outcome variables) were dichotomised with category 1 for meeting the variable criteria (1 = adequate) and category 0 for not meeting the variable criteria (0 = not adequate). Descriptive statistics were presented using means, standard deviations (SDs), frequencies and proportions. Multivariate logistic regression analysis was employed with AHDDS and ACDDS as dependent variables against independent variables to identify factors associated with dietary diversity adequacy (Kiboi et al., 2017; Mekuria et al., 2017; Workicho et al., 2016). To account for the design effect, the logistic regression analysis included a categorical variable (place of residence) that indicates to which IDP a household belongs. Bivariate logistic regression analysis was first undertaken for each explanatory variable with the outcome binary variables AHDDS and ACDDS. Variables with a *p* ≤ 0.2 in the bivariate logistic regression analysis (crude odds ratio [COR]) were included in the multivariate logistic regression analysis (adjusted odds ratio [AOR]). Results from the logistic regression analysis are presented as COR and AORs with 95% confidence intervals. Statistical significance was declared if the *p* value was <0.05. The data were analysed using STATA version 16.

## 3 RESULTS

### 3.1 Characteristics of households

Overall, 1655 households and 2370 children were included in the study. The characteristics of the households are presented in Table 1. Most female main caregivers were married (94.3%) and could not read or write (87.2%). Approximately 25% of the households comprised at least six members. Nearly half of the female main caregivers were aged 25–34 years, and two‐thirds of the respondents had not received ANC during their last pregnancy.

**Table 1:** Social characteristics of households (*n* = 1655).

Variables	*n*	%
Place of residence		
Baidoa	946	57.2
Dharkanley	573	34.6
Dayniile	136	8.2
Female main caregiver age		
15–24	504	30.5
25–34	753	45.5
35–44	339	20.5
>45	59	3.5
Marital status of female main caregiver		
Married	1561	94.3
Divorced/separated	94	5.70
Wealth index (quintiles)		
Poorest (first quantile)	330	19.9
Poor (second quantile)	332	20.0
Middle (third quantile)	308	18.6
Richer (fourth quantile)	345	21.0
Richest (fifth quantile)	340	20.5
Household size		
<6	1235	74.6
≥6	420	25.4
Number of children <5 years		
<2	1500	90.6
≥2	155	9.40
Literacy status		
Can read and write	212	12.8
Cannot read and write	1443	87.2
Decision‐making about daily household purchase needs		
Female main caregiver	702	42.4
Husband	496	30.0
Joint decision‐making	447	27.0
Someone else	10	0.60

### 3.2 Household and child dietary diversity

The mean number of food groups per day on the HDDS in Baidoa, Dharkanley and Dayniile IDP households were (mean ± SD) 8.2 ± 2.0, 7.0 ± 1.4 and 6.5 ± 1.6, respectively (Table 2). The proportion of households with AHDDS was 95.8%, 96.9% and 89.0% in Baidoa, Dharkanley and Dayniile, respectively, and the total AHDDS was 95.6%. The mean CDDS scores among IDP households in Baidoa, Dharkanley and Dayniile were 4.9 ± 2.2, 2.9 ± 1.4 and 3.2 ± 0.9, respectively. ACDDS was achieved in 63.5%, 8.5% and 38.3% of households in Baidoa, Dharkanley and Dayniile, respectively (Table 2), and the total ACDDS was 43.3%.

**Table 2:** Dietary diversity scores in households (*N* = 1655) and in children (*N* = 2370).

Variables	Baidoa	Dharkanley	Dayniile
Number of households/number of children	946/1300	573/809	136/261
HDDS, mean ± SD	8.20 ± 2.04	7.01 ± 1.36	6.50 ± 1.63
AHDDS, *n* (%)	906 (95.8)	555 (96.9)	121 (89.0)
CDDS, mean ± SD	4.90 ± 2.16	2.90 ± 1.35	3.20 ± 0.95
ACDDS, *n* (%)	825 (63.5)	69 (8.5)	14 (5.4)

Abbreviations: ACDDS, adequate child dietary diversity score; AHDDS, adequate household dietary diversity score; CDDS, child dietary diversity score; HDDS, household dietary diversity score.

**Figure 1:**
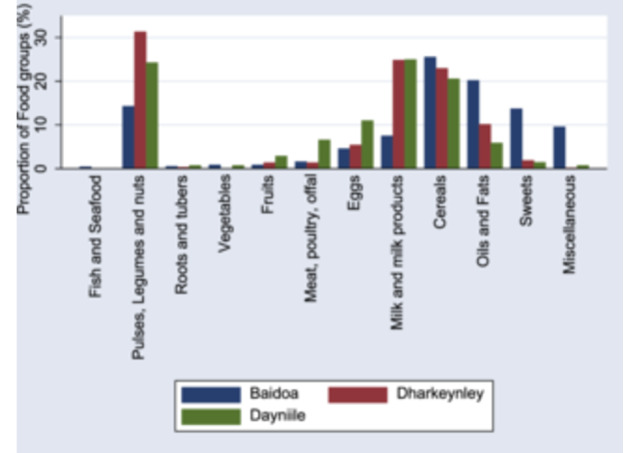
Proportion of household dietary food groups in each district.

Furthermore, Figure 1 shows that the most consumed food groups were pulses, legumes and nuts followed by milk and milk products and cereals.

### 3.3 Factors associated with AHDDS

Findings from the multivariate logistic regression analysis showed that place of residence was a significant factor for AHDDS; households in Dayniile and Dharkanley were less likely to consume with adequate dietary diversity compared to those in Baidoa (Table 3). Households with more than six members and/or who were wealthier had greater odds of consuming with adequate dietary diversity. Households with caregivers who had received ANC and/or had practiced timely initiation of complementary feeding were significantly more likely to achieve adequate dietary diversity scores. The analysis also showed that joint decision‐making between the male and the female caregivers in the household was significantly positive for an adequate dietary diversity score compared to those households in which decisions were made only by females (Table 3).

**Table 3:** Logistic regression analysis showing the COR and AOR for investigated factors on AHDDS (*n* = 1582).

	AHDDS			
Factors	*n*	%	COR (95% CI)	AOR (95% CI)
Place of residence				
Baidoa	906	(95.8)	1	1
Dharkanley	555	(96.9)	0.05 (0.04, 0.07)***	0.20 (0.07, 0.58)***
Dayniile	121	(89.0)	0.03 (0.02, 0.05)***	0.03 (0.01, 0.17)***
Literacy status				
Can read and write	203	(95.7)	1	1
Cannot read and write	1379	(96.0)	1.03 (0.51, 2.10)	0.80 (0.36, 1.70)
Marital status of female main caregiver				
Divorced/separated	90	(95.7)	0.96 (0.34, 2.69)	0.77 (0.24, 2.52)
Married	1492	(95.6)	1	1
Number of children < 5 years				
<2	1434	(95.6)	1	1
≥2	148	(95.5)	0.97 (0.44, 1.16)	1.01 (0.42, 2.49)
Household size				
<6	1170	(94.7)	1	1
≥6	412	(98.1)	2.86 (1.36, 6.04)***	2.56 (1.14, 5.79)**
ANC attendance				
Yes	604	(97.1)	1	1
No	978	(94.7)	0.52 (0.31, 0.91)**	0.49 (0.27, 0.89)**
Female main caregiver age (years)				
14–24	478	(94.8)	1	1
25–34	720	(95.6)	1.20 (0.70, 2.01)	1.16 (0.65, 2.08)
35–44	325	(95.9)	1.30 (0.65, 2.45)	0.95 (0.45, 1.99)
>45	59	(100.0)	–	–
Wealth Index (quintiles)				
Poorest (first quantile)	304	(92.1)	0.68 (0.36, 1.29)	0.17 (0.04, 0.78)**
Poor (second quantile)	316	(95.2)	1.18 (0.58, 2.38)	0.19 (0.04, 0.86)**
Middle (third quantile)	302	(98.1)	2.85 (1.11, 7.33)**	1.58 (0.31, 8.14)
Richer (fourth quantile)	337	(97.7)	2.85 (1.21, 6.71)**	1.15 (0.25, 5.39)
Richest (fifth quantile)	323	(95.0)	1	1
Timely initiation of complementary feeding				
Yes	737	(97.9)	1	1
No	845	(93.7)	0.32 (1.83, 0.56)***	0.29 (0.16,0.53)***
Decision‐making about daily household purchase needs				
Female main caregiver	666	(94.9)	1	1
Husband	468	(94.35)	0.90 (0.54, 1.50)	0.91 (0.47, 1.77)
Joint decision‐making	440	(98.43)	3.39 (1.49, 7.70)***	2.87 (1.20, 6.83)**
Someone else	8	(80.00)	0.21 (0.44, 1.05)	0.37 (0.06, 2.44)

Abbreviations: AHDDS, adequate household dietary diversity score; ANC, antenatal care; AOR, adjusted odds ratio; CI, confidence interval; COR, crude odds ratio.

** *p* < 0.01

*** *p* < 0.001.

### 3.4 Factors associated with ACDDS

Children who lived in Dharkanley and Dayniile were less likely to have an ACDDS compared with those in Baidoa. Children aged 12–59 months were more likely to consume with adequate dietary diversity compared to children aged 6–11 months. Children who lived with their main caregiver were also more likely to consume with adequate dietary diversity than those who did not. Furthermore, children who had received a deworming tablet (a proxy measure of recovery from diarrhoea) and/or were fed with RUTF (a proxy measure of malnutrition recovery) more frequently had a higher ACDDS than their counterparts. Children who had been sick in the 2 weeks before the survey were less likely to consume with adequate dietary diversity than those who had not been sick. Finally, the children whose caregivers attended ANC were significantly more likely to have an adequate dietary diversity score compared to those children whose caregivers did not attend ANC (Table 4).

**Table 4:** Logistic regression analysis showing the COR and AOR for investigated child health and nutrition factors on ACDDS (*n* = 908).

	ACDDS			
Factors	*n*	%	COR (95% CI)	AOR (95% CI)
Place of residence				
Baidoa	825	(63.5)	1	1
Dharkanley	69	(8.5)	0.05 (0.04, 0.07)	0.02 (0.02, 0.04)***
Dayniile	14	(5.4)	0.03 (0.02, 0.05)	0.02 (0.14, 0.05)***
Child's age (months)				
6–11	156	(29.1)	1	1
12–23	213	(35.9)	1.36 (1.06, 1.75)**	1.54 (1.48, 2.51)**
24–35	245	(44.2)	1.93 (1.59, 2.48)***	2.24 (1.56, 3.18)***
36–47	237	(45.6)	2.03 (1.58, 2.62)***	2.02 (1.41, 2.86)***
48–59	57	(34.3)	1.27 (0.87, 1.84)	1.93 (1.11, 3.34)**
Child lives with female main caregiver				
Yes	772	(42.8)	1	1
No	136	(24.0)	0.42 (0.34, 0.52)***	0.16 (0.12, 0.21)***
Child has received deworming tablet				
Yes	536	(42.0)	1	1
No	372	(34.0)	0.71 (0.60, 0.84)***	0.55 (0.42, 0.73)***
Child's admission to CMAM in the past 3 months				
Yes	335	(42.4)	1	1
No	573	(36.3)	0.77 (0.65, 0.92)***	0.98 (0.73, 1.33)
Child has been fed with RUTF				
Yes	458	(58.9)	1	1
No	450	(28.3)	0.27 (0.23, 0.33)***	0.52 (0.39, 0.67)***
Child has been sick in the last 14 days				
Yes	375	(36.0)	1	1
No	533	(40.0)	1.19 (1.01, 1.41)*	1.78 (1.36, 2.33)***
Children whose caregivers attended ANC				
Yes	772	(85.0)	1	1
No	136	(15.0)	0.83 (0.70, 0,99)**	0.77 (0.64, 0.92)***

Abbreviations: ACDDS, adequate child dietary diversity score; ANC, antenatal care; AOR, adjusted odds ratio; CI, confidence interval; CMAM, community‐based management of acute malnutrition; COR, crude odds ratio; RUTF, ready‐to‐use therapeutic food.

* *p* < 0.05

** *p* < 0.01

*** *p* < 0.001.

## 4 DISCUSSION

This study is one of very few to explore household and child dietary diversity in IDP camps and its associated factors in Somalia. The findings provide valuable insights into household and child dietary diversity in the studied population, which included 1655 households and 2370 children, providing a substantial sample size for analysis. The study highlights disparities in dietary diversity within IDP camps in Southern Somalia, showing high household dietary diversity but low child dietary diversity. This discrepancy might be due to factors like larger household sizes and wealth impacting dietary choices differently for adults and children (Codjoe et al., 2016; Curtin et al., 2024).

One of our key findings was that the proportion of households achieving an AHDDS was high across all locations, while the proportion of children with an ACDDS was low in all settings, especially in Dharkanley. Higher AHDDS and lower ACDDS were also found in similar settings (Federica Di Marcantonio et al., 2020). This suggests that a majority of households but not all children were able to achieve a diverse diet. Furthermore, a multifaceted approach involving multiple sectors, such as health education, nutrition counselling, ANC, maternal dietary diversity, women's empowerment, ANC and cash transfer, was shown to be most effective in improving CDDS (Hoque, 2023; Kurdi, 2021). Tailoring interventions to the specific cultural and socioeconomic contexts of the community is essential for this success (Rees et al., 2022; Reinbott et al., 2016).

Household size and wealth were identified as important factors contributing to dietary diversity, with larger households being more likely to have an AHDDS. This finding is consistent with those of several studies conducted in other regions (Agrawal et al., 2019; Marinda et al., 2018). Although larger household size and higher wealth increase the demands on the adults in the household and decrease their ability to overcome obstacles that require monetary input, some studies have shown that larger household size is positively associated with a higher dietary diversity score (Christian et al., 2019; Cordero‐Ahiman et al., 2021). This higher score may be due to more members contributing to household dietary diversity in larger households (Jones et al., 2014), which partly reflects the limited robustness of the measure of wealth. However, other evidence has shown that household size may be negatively associated with food security (Kihiu & Amuakwa‐Mensah, 2021). In Somalia, most families have numerous children who are engaged in income‐generating activities, which may in turn allow families to afford the required food groups. It is important to note that improving dietary diversity in larger households requires a combination of creative strategies, informed decision‐making and community engagement. Tailoring these strategies to community‐specific cultural, economic and social contexts may maximise their effectiveness (Bellon et al., 2016).

A concerning finding was that two‐thirds of the respondents had not received ANC during their last pregnancy, potentially indicating limited access to health care services. The findings showed that ANC was positively associated with higher dietary diversity scores. ANC visits serve as a valuable opportunity for health care providers to impart crucial nutritional knowledge to expectant mothers and to emphasise the significance of proper nutrition during pregnancy (Katenga‐Kaunda et al., 2021). Additionally, our findings demonstrated that children who consumed deworming tablets and RUTF had a higher chance of an ACDDS. Mothers who attended ANC sessions in which they received guidance or health education may have contributed to this outcome (Kayemba et al., 2023). However, this is simply a hypothesis to explore as our study did not explicitly investigate this relationship.

Joint decision‐making between male and female caregivers was also significantly associated with adequate dietary diversity, highlighting the importance of shared decision‐making in promoting a diverse diet. Previous research has similarly shown that a lower HDDS occurs when decision‐making is left to the female household head only (Ng'endo et al., 2016). A study in Malawi found that households in which shared decision‐making activities occurred had higher HDDS (Ragasa et al., 2019). Therefore, our results suggest that because shared decision‐making improves dietary diversity scores, interventions and projects focusing on dietary intake should consider shared decision‐making from a gender perspective. This shared responsibility can lead to increased motivation to adopt a diverse diet as the female and male caregivers work towards common health goals (Jones et al., 2014).

The presence of the child's main caregiver was also positively associated with CDDS, while children who were separated from their main caregiver or orphaned had lower dietary diversity than nonorphaned children. The findings suggest that children separated from their main caregiver or orphaned might be at a higher risk of inadequate nutrient intake due to limited access to diverse food sources and the absence of parental guidance in meal planning. This situation may be exacerbated by the vast displacement of IDPs due to droughts and conflicts. The separation can result in far‐reaching implications that can lead to stunted growth, compromised cognitive development and increased susceptibility to infections and diseases (Ali et al., 2018; Thorne‐Lyman et al., 2010).

Despite its contributions, this study has some limitations. The study used a 24‐h recall method, which provides data about a one‐time phenomenon but does not account for dietary habits that may be affected by seasonal variations. Furthermore, the study did not measure mean dietary adequacy, minimum acceptable diet or the mean density of nutrients. Social desirability bias may also be relevant to consider in this context; it is possible that households that have previously received food vouchers or cash assistance may overestimate their food consumption to demonstrate that they are effectively utilising the support provided. Conversely, some households may underestimate their consumption to receive additional assistance. Finally, purposively selecting districts may limit the generalisation of the results to other settings.

## 5 CONCLUSION: PRACTICE AND POLICY IMPLICATIONS

In conclusion, this study, with a sample size of 1655 households and 2370 children, offers valuable insights into household and child dietary diversity within IDP camps in Somalia. The findings reveal that while a considerable proportion of households achieve adequate dietary diversity, adequate child dietary diversity, measured by CDDS, is low, especially in certain locations, such as Dharkanley. Notably, household size and wealth play significant roles in achieving dietary diversity, with larger households being more likely to have sufficient diversity. However, the identified association between larger household size and lower CDDS suggests the need for policy interventions that address the unique challenges faced by larger families in providing diverse and nutritious diets for their children. One potential policy implication is the development of targeted nutrition education campaigns. These campaigns could be tailored to the specific needs of larger households, providing practical guidance on optimising available resources to ensure that children receive a variety of nutrient‐rich foods. Moreover, policies could facilitate peer support and knowledge sharing among caregivers in larger households and consider economic support mechanisms that alleviate the financial burden on larger households, such as cash transfers (Abdullahi et al., 2023).

The study highlights the positive impact of receiving ANC on dietary diversity scores. The consumption of deworming tablets and RUTF was also shown to enhance CDDS, underscoring the importance of health interventions. Policymakers could prioritise the integration of nutrition education within ANC programmes by providing expectant mothers with comprehensive information on optimal maternal nutrition and its direct influence on their child's dietary diversity; such programmes can empower caregivers to make informed choices that positively impact their child's nutritional outcomes.

Overall, the policy implications resulting from our findings can guide the development and implementation of targeted interventions and programmes to improve household and child dietary diversity, contributing to better health outcomes in settings similar to those of IDP camps in Somalia.

## AUTHOR CONTRIBUTIONS

Mohamed K. Ali and Munshi Sulaiman contributed in conceptualisation. Mohamed K. Ali, Renée Flacking, Lars Berglund, Munshi Sulaiman and Fatumo Osman contributed in methodology. Mohamed K. Ali contributed in formal analysis. Mohamed K. Ali, Renée Flacking, Munshi Sulaiman, Lars Berglund and Fatumo Osman contributed in investigation. Munshi Sulaiman contributed in resources. Mohamed K. Ali, Munshi Sulaiman and Lars Berglund contributed in data curation. Mohamed K. Ali contributed in writing (original draft prepartaion). Mohamed K. Ali, Renée Flacking, Munshi Sulaiman and Fatumo Osman contributed in writing (review and editing). Mohamed K. Ali contributed in visualisation. Renée Flacking, Fatumo Osman and Munshi Sulaiman contributed in supervision. Mohamed K. Ali contributed in project adminstration. All authors have read and agreed to the published version of the manuscript.

## CONFLICT OF INTEREST STATEMENT

The authors declare no conflict of interest.

## Data Availability

Restrictions apply to the availability of these data, which were used under license for this study. Data are available from the author(s) with the permission of [third party].
